# Male partner involvement in birth preparedness, complication readiness and obstetric emergencies in Sub-Saharan Africa: a scoping review

**DOI:** 10.1186/s12884-021-03606-x

**Published:** 2021-02-12

**Authors:** Faye Forbes, Karen Wynter, Berihun M. Zeleke, Jane Fisher

**Affiliations:** 1 Global and Women’s Health, School of Public Health and Preventive Medicine, Monash University, 553 St Kilda Rd, Melbourne, Victoria 3004 Australia; 2 Deakin University School of Nursing and Midwifery, Geelong, Victoria Australia; 3 Centre for Quality and Patient Safety Research – Western Health Partnership, St Albans, Victoria Australia; 4 School of Public Health and Preventive Medicine, Monash University, 553 St Kilda Rd, Melbourne, Victoria 3004 Australia; 5 Institute of Public Health, College of Medicine and Health Sciences, University of Gondar, Gondar, Ethiopia

**Keywords:** Birth preparedness and complication readiness, Male partner, fathers, Male involvement, Sub-Sahara Africa

## Abstract

**Background:**

Maternal mortality remains a pressing concern across Sub-Sahara Africa. The ‘Three Delays Model’ suggests that maternal deaths are a consequence of delays in: seeking care, reaching medical care and receiving care. Birth Preparedness and Complication Readiness (BPCR) refers to a plan organised during pregnancy in preparation for a normal birth and in case of complications. Male partners in many Sub-Saharan African communities could play a pivotal role in a woman’s ability to prepare for birth and respond to obstetric complications. This review aimed identify: the extent and quality of research performed on the topic of male partner involvement in BPCR in Sub-Saharan Africa; the degree to which populations and geographic areas are represented; how male partner involvement has been conceptualized; how male partners response to obstetric complications has been conceptualised; how the variation in male partners involvement has been measured and if any interventions have been performed.

**Methods:**

In this scoping review, articles were identified through a systematic search of databases MEDLINE, EMBASE and Maternity and Infant Care and a manual scan of relevant papers, journals and websites. All authors contributed to the screening process and a quality assessment using the Kmet checklist. The PRISMA checking list for Scoping Reviews was used to guide the search, data charting and reporting of the review The protocol was registered with PROSPERO (ID: CRD42019126263).

**Results:**

Thirty-five articles met inclusion criteria, reporting: 13 qualitative, 13 cross-sectional, 5 mixed method and 4 intervention studies. Data were contributed by approximately 14,550 participants (numbers were not always reported for focus groups) including: women who were pregnant or who had experienced pregnancy or childbirth within the previous 3 years, their male partners and key informants such as health workers and community leaders.

**Conclusions:**

The diversity of study designs, aims and source countries in this body of literature reflects an emerging stage of research; as a result, the review yielded strong evidence in some areas and gaps in others. Male partner’s involvement in BPCR and responding to obstetric emergencies can be conceptualised as being centrally involved in responding to complications and having some role in preparing for birth through their position in the chain of decisions and provision of logistic support. However, their knowledge of pregnancy complications and level of preparation for birth is low, suggesting they are making decisions without being fully informed. There is limited evidence on interventions to improve their knowledge. Future research efforts should be focused on producing standardised, culturally appropriate, higher level evidence.

**Supplementary Information:**

The online version contains supplementary material available at 10.1186/s12884-021-03606-x.

## Background

Globally, approximately 830 women die each day due to pregnancy complications, 66% of these deaths occur in Sub-Saharan Africa [1]. Most of these deaths could be prevented by timely access to medical support during pregnancy, labour and the postnatal period. According to the ‘three delays’ model, maternal deaths are frequently related to a delay in: 1) seeking care, 2) reaching medical care and 3) receiving adequately skilled care once at a facility [2, 3].

Advanced preparation for childbirth by women who are pregnant and their families, is one method of reducing life threatening delays in receiving care during birth [4, 5]. Birth preparedness and complication readiness (BPCR) refers to a plan, organised during pregnancy in preparation for a normal delivery and in case of complications [4, 5]. BPCR includes: identifying a skilled birth attendant, identifying the nearest facility, saving money for the birth costs, organising transport in advance, identifying a birth companion, identifying a potential blood donor and knowing the signs of complications [6]. In 2015, the World Health Organisation (WHO) endorsed the use of BPCR interventions stating that, ‘BPCR interventions are recommended to increase the use of skilled care at birth and to increase the timely use of facility care for obstetric and newborn complications’ [7].

Data from the Demographic Health Survey (DHS) indicates that male partners in many parts of Sub-Saharan Africa are key decision makers in many families, including decisions about maternal health [8]. It is plausible that male partners could play a pivotal role in a woman’s ability to prepare for birth and respond to obstetric complications. Male involvement in reproductive health was first agreed to be an international priority at the International Conference on Population and Development (UNFPA 1994) in Cairo. Since then countries throughout Sub-Saharan Africa have, to varying degrees, recognised the importance of including male partners in reproductive healthcare [9].

Most research and evidence syntheses about BPCR has been conducted from among women [4, 5, 10]. Although there is some evidence about the level of involvement and the role of men in BPCR and responding to obstetric complications, it is yet to be synthesised in systematically conducted reviews.

The literature contains significant diversity in the way male partner involvement has been conceptualised, the types of questions that have been asked, the research methods employed and the results. The lack of uniformity amongst the evidence prevents a systematic review being performed at this stage, and suggests that a scoping review is appropriate to determine the extent of research and to map, summarise and identify gaps in the evidence.

### Objectives

This review aimed identify: the extent and quality of research performed on the topic of male partner involvement in BPCR in Sub-Saharan Africa; the degree to which populations and geographic areas are represented; how male partner involvement has been conceptualized; how male partners response to obstetric complications has been conceptualised; how the variation in male partners involvement has been measured and if any interventions have been performed.

## Methods

This review adhered to Cochrane Consumers and Communication review group guidelines, JBI Manual for Evidence Synthesis Chapter 11: Scoping Reviews and the PRISMA checking list for Scoping Reviews to guide the search, data charting and reporting of the review [11–13]. The review was registered with PROSPERO (ID: CRD42019126263).

### Definitions

BPCR was defined as planning and/or organising during pregnancy in preparation for a normal delivery or in case of complications. The BPCR actions included saving money for birth; identifying transport; identifying the birth location; knowing the signs of pregnancy complications; identifying a skilled birth attendant, identifying someone to donate blood. Complications were defined as: Immediate, life threatening pregnancy or labour complications.

Male partner involvement was defined as a male partner’s attitudes, behaviours or experiences in relation to BPCR or obstetric emergencies.

### Information sources and search strategy

Databases (EMBASE, Ovid MEDLINE and Maternity and Infant Health) were searched for records in English. The review topic was divided into the following concepts 1) Male involvement, 2) Birth preparedness and complication readiness, 3) Obstetric emergencies and 4) Sub-Saharan Africa. Appropriate MeSH terms and truncated key words were adopted for each concept. Boolean operators AND /OR were used to link concepts and associated terms in the following way: (male involvement) AND (birth preparedness complication readiness OR obstetric emergencies) AND (Sub-Saharan Africa) (see supplementary table 1 for detailed search strategy). The authors also undertook a manual search of the reference lists in relevant publications, journals and websites for additional studies.

### Eligibility criteria

The following eligibility criteria were adopted: peer-reviewed research; humans; English language; Sub-Saharan Africa; primary research; male participants or other participants reporting on male partner involvement (men’s attitudes, behaviours or experiences) and BPCR (at least one indicator) OR Pregnancy/ birth complications in aims, primary outcome (quantitative studies) or main theme (qualitative studies). All studies which met criteria after 2005 and until the final search date November 2019 were included.

### Study selection

Study screening was performed in Covidence [14] with participation from all four reviewers. Studies were double screened at 1) title and abstract stage and 2) full text review stage. Differences of opinion at any stage were resolved by discussion between the author group.

### Scope of the review

The search strategy specifically sought studies reporting on male partner involvement in birth preparedness and complication readiness or obstetric emergencies. The findings were analysed to identify male partner attitudes, behaviours and experiences in relation to BPCR and response to obstetric complications as defined by the review aims. Results regarding only attendance at antenatal care, the presence of skilled birth attendance and general involvement of men during pregnancy, childbirth and in maternal and child healthcare were not included. Male involvement in post-partum complications was reported if the results were available in the included articles, however the search strategy did not specifically search post-partum complications.

### Data charting process and data items

At least two authors extracted data independently for each paper. Data charting fields included; authors (date); country; study design; aim; inclusion criteria; sample characteristics; number of participants; recruitment strategy; data source; analysis and key findings related to review.

### Critical appraisal of sources of evidence

In order to evaluate the quality of research available on this topic, in accordance with the reviews aims a critical appraisal of the literature was performed using the Kmet checklist [15]. Separate checklists were used to evaluate research deemed primarily qualitative and primarily quantitative. Individual criteria were scored 0–2 and a final score was produced (sum of scores as a proportion of potential maximum score). All papers were assessed by two authors and differences of opinion resolved via discussion until a consensus was achieved.

## Results

**Fig. 1:**
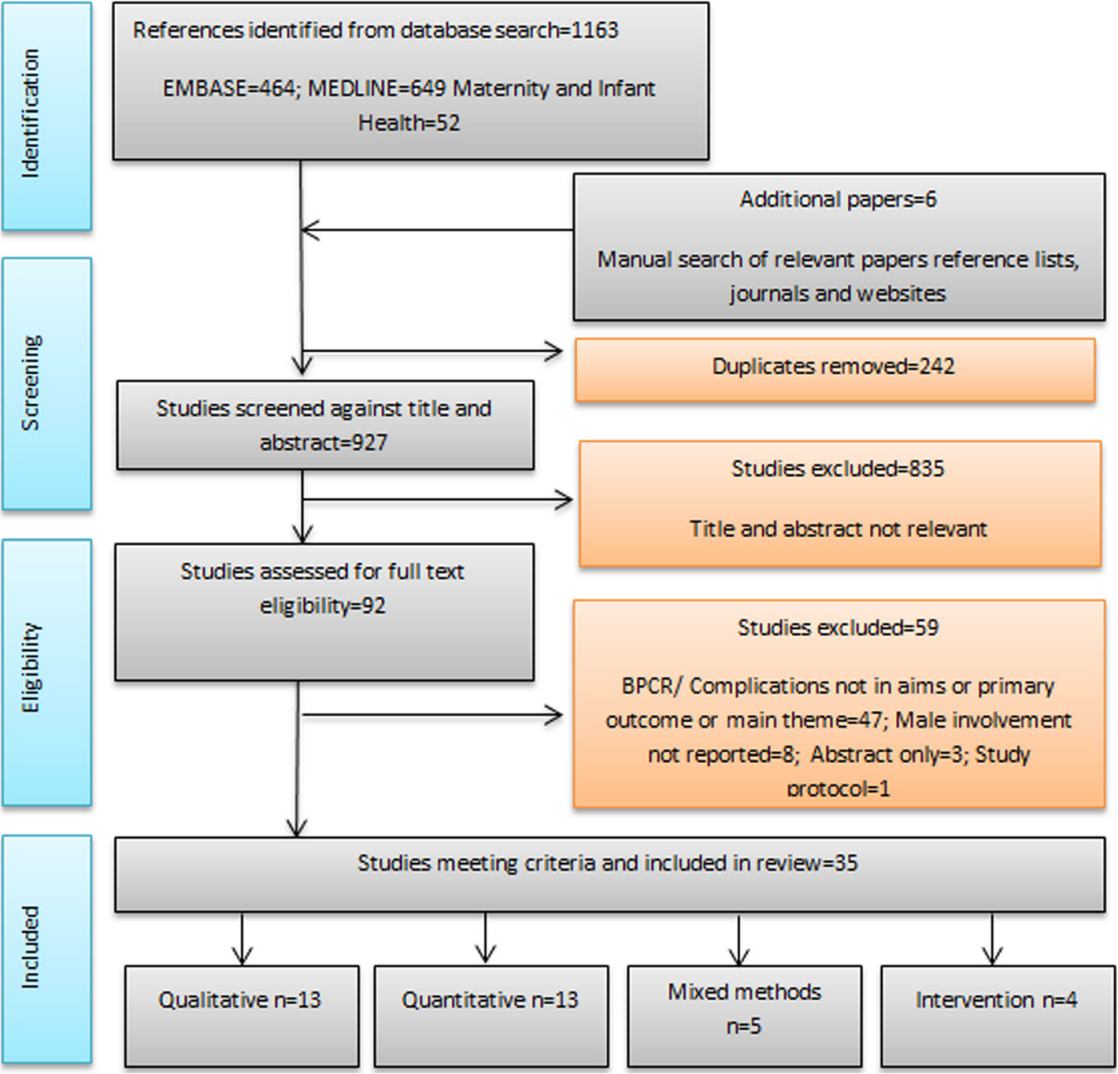
Flow chart of study selection

The selection process for all sources of evidence included is provided in a flow chart (Fig. 1).

The study characteristics and results as they relate to the review aims, are provided for quantitative, qualitative and mixed-method studies in Table 1 and intervention studies in Table 2.

**Table 1:** Quantitative, qualitative and mixed-method studies included

Authors (date)	Aim	Inclusion criteria and sample (n)	Recruitment strategy	Data source & analysis	Findings	QA
**Qualitative research 90 FGD and 393 IDI/SSI**						
Burkina Faso						
Some, Sombie&Meda (2013) [16]	To examine how decisions are made for maternal care in rural Burkina Faso	Women aged 15–49 years who had recently given birth and had or had not used a facility (8 FGD and 30 IDI)	Recruited using snowball technique	- FGD, IDI -Topic guide not reported -Thematic analysis	-MP only involved in complications -Men in control of money -Women need permission to leave home -MP decision influenced by women and women’s relatives	.60*
Ghana						
Ganle & Dery (2015) [17]	To describe men’s perceptions, attitudes and involvement in maternal healthcare and how women navigate maternal healthcare	Men whose wives were pregnant or lactating, and community leaders (12 FGD, 50 IDI with men and spouses, 30 IDI with community leaders)	Purposive sampling to capture diversity of social and health situations representative of region Recruitment via community and religious leaders	- FGD, IDI -Topic guides: 1) FGDs with men: SBA and barriers/enablers men’s involvement in MCH 2) IDI with women: women’s experiences with men’s involvement in MCH 3) IDI key informants: male involvement in MCH -Thematic analysis	-MP only involved if complications occurred	.80*
Story, Barrington, Fordham, Sodzi-Tettey, Barker & Singh (2016) [18]	To explore the various types of male involvement and health facility accommodation during obstetric emergencies	Women who experienced severe birth complications and their partners (8 FGD with 59 stakeholders, IDI with 21 women, 18 men and 6 key informants))	Purposive sampling for a range of health facilities and couples that had experienced complications	- FGD, IDI -Topic guide: personal experiences of childbirth and birth complications. Women explained the male partner’s role during the experience and male partners were asked about the woman’s crisis -Inductive analytic approach, with comparison between men and women	-Women not involved in decisions about their healthcare -If complications occur chain of discussion is: woman-man-his mother-man then decision is made. − 25% MP not involved at all in complications. −67% MP instrumentally involved (transport and fees) or emotionally involved (prayers) during complications -MP often did not attend facility -HW said it took vital time to contact MP for money/decisions -HW mixed attitudes towards MP	.80*
Aborigo, Reidpath, Oduro&Allotey (2018) [19]	To explore men’s reluctance to be involved in MCH	Opinion leaders (majority male): chiefs, elders, assemblymen, leaders of women’s groups Healthcare workers (10 FGD with 120 participants, 16 IDI)	Purposive selection where community chiefs asked 10–12 opinion leaders	- FGD, IDI -Topic guide: Opinions on the lack of support for women and delays in receiving care -Thematic analysis	-MP save money -Women wanted MP more involved in BPCR -MP discuss pregnancy care with TBA -Women not allowed to access care independently	.60*
Kenya						
Brubaker, Nelson, McPherson, Ahn, Oguttu& Burke (2016) [20]	To understand the role of men in MCH	Men, women and community health workers (18 FGD with 134 participants)	Purposive sampling for facility and home birth until saturation achieved Recruited by health workers	- FGD -Topic guide: birth experiences, preparations, individual and community expectations of male roles, obstacles to male involvement -Thematic analysis, inductive/ set-list	-MP make MCH decisions because they make the money -MP significant in complications -Men have poor knowledge of MCH	.85*
Malawi						
Manda-Taylor, Mwale, Phiri, Walsh, Matthews, Brugha& Byrne (2017) [21]	To explore the role of men in MCH	Women using/ not using MCH services, vulnerable women, household members such as husbands and women (20 FGD, 85 IDI)	Purposive sampling based on socio-demographic characteristics for diversity. Snowball technique to recruit hard to reach participants	- FGD, IDI -Topic guide: formal and informal community system enablers and barriers to using MCH services -Thematic analysis	-MP role to provide money	.80*
Aarnio, Kulmala& Olsson (2018) [22]	To document husbands’ role in decision-making and healthcare seeking in cases of pregnancy complications	Husbands and wives who had experienced complications within past 5 years Key informants: Village chief and wife, mother and uncle head of clan, TBA (SSI with 12 husbands, 12 wives, 6 key informants)	Purposive sampling with assistance of two village headmen	- SSI -Semi-structured interview guide: experiences of complications, perceptions of husband’s role in decision making and seeking care for complications - Thematic analysis based on Bourdieu’s concepts of “capital” and “field”	-MPs economic and symbolic capital in healthcare decisions during complications -Role attributed to their position as father, main income earner and head of the household -Lack of money is only reason to deny women access to healthcare -MPs have limited knowledge of MCH	.85*
Tanzania						
Pembe, Urassa, Darf, Carlsted& Olsson (2008) [23]	To describe perceptions of maternal referrals in a rural district in Tanzania	Health workers: midwives, MCH aide, nurse assistants Community groups: young men and women, old men and women (10 FGD with 11 health workers and 85 community members)	Purposive sampling for representation of all hamlets Recruitment via village chairperson	- FGD -Topic guides: Where the community seeks care; what the danger signs are; referral decision processes; factors surrounding referrals -Content analysis	-Women have limited influence on decisions during complications -MP and relatives are the key decision makers	.65*
Moshi &Nyamhang (2017) [9]	To explore the socio-cultural barriers to health facility birth and SBA among parents choosing home birth in rural Tanzania	Matched couples: partnered men and women whose youngest child had been delivered at home less than 12 months ago (4 FGD, 32 IDI)	Purposive sampling for women who had experienced home birth via village head The same participants were used for both FGD and IDI	- FGD, IDI -Topic guides: 1) FGD: general socio-cultural barriers in the community 2) IDI: personal experiences with home childbirth -Thematic analysis and triangulation FGD and IDI	-MP provide transport -MP view pregnancy and childbirth as a natural and risk-free process	.85*
Uganda						
Mbalinda et al. (2015) [24]	To understand how obstetric complications are perceived by MPs	MPs of women who experienced near-miss event (25 IDI)	Purposive sampling	- IDI -Explored partners’ experiences and perceptions of women’s recovery from a near-miss event. -Thematic analysis	-MPs experience intense fear and worry, financial loss, newborn death and loss of time while in hospital -Excluded from healthcare discussions and decisions -Support from social network -Isolation and ongoing distress	.85*
Nansubuga&Ayiga. (2015) [25]	To examine the roles played by MP after near-miss obstetric complications	MP of women who experienced a maternal near-miss (10 IDI)	Purposive sampling from a large cross-sectional study sample (randomly selected)	-IDIs -Content analysis	- MP involved in managing household level response to life-threatening complications: intramuscular medication, oral medication and massage -Decision making -Financial support -Social support -Transport	.65*
Kaye, Kakaire, Nakimuli, Osinde, ScoviaMbalinda&Kakande (2014) [26]	To understand MP involvement in childbirth complications	Male partners of women who developed obstetric complications and were admitted to hospital (16 IDI)	Recruited via women admitted to hospital	- IDI -Thematic analysis	-Ideally fathers are involved and supportive -MP willing to support partners but hampered by health system -No clear roles in the hospital environment -Excluded from decisions.	.80*
Zambia						
Sialubanje Massar, Kirch, van der Pijl, Hamer & Ruiter (2016) [27]	To explore men’s beliefs and experiences regarding maternity waiting homes	Husbands or partners (aged 18–50 years) of women who attended a health centre with a waiting home. Wife reproductive age, given birth in last year (24 IDI)	Purposive sampling for experience of waiting homes and range of districts, health centres and families Health officers at waiting homes informed women of study, then women asked husbands to be involved	- IDI -Topic guide: Husbands’ perceived benefits and barriers, decision making process and their roles in their wives’ use of waiting homes -Short demographic questionnaire -Thematic analysis and demographic statistics	-MP plan for birth finances -MP main roles in pregnancy and birth: decision maker for waiting home; money for food and transport; clothes and items for newborn; finding someone to take care of children -Decisions not unilateral, men and women discuss issues together	.80*
**Quantitative research** ***n*** **= 5942**						
Ethiopia						
Baraki et al. (2019) [28]	To assess MP involvement in BPCR	Men whose wives had an infant aged up to 12 months in a community household (406)	Randomly selected lottery sample	Cross-sectional observational study -Interviewer administered standard JHPIEGO questionnaire -Multivariate analysis	See Table 3	.72†
GebrehiwotWeldearegay (2015) [29]	To assess MP involvement in BPCR	Men whose wives had an infant less than 12 months. Men separated or with critically ill children excluded (398)	Randomly selected sample	- Cross-sectional observational study -Interviewer administered standard JHPIEGO questionnaire -Multivariate analysis	See Table 3	.86†
Mersha (2018) [30]	To determine men’s level of knowledge about obstetric danger signs and level of BPCR	Men whose wife gave birth within past 2 years (824)	Multistage cluster sampling procedure selected 4 districts from 19, all households within catchment areas with eligible men invited	- Cross-sectional observational study - Standardised structured JHPIEGO questionnaire adapted for Ethiopia: Socio demographics, knowledge of danger signs, BPCR - Frequencies and %. Bivariate logistic regression and multivariate regression.	See Table 3	1. †
Tadesse, Boltena & Asamoah (2018) [31]	To assess husbands’ level of participation in BPCR and associated factors	Husbands of pregnant woman and nursing mothers Husband age 20–50. Religion: majority versions of Christianity Occupation: largest groups merchant, labourer, government employed (592)	Multistage sampling technique. Eight districts randomly selected from Wolaita town. Sampling frame of households in which a pregnant woman was living known from ANC registration. Systematic random sampling of 607 households with a woman registered for ANC.	- Cross-sectional observational study - Standardised structured BPCR questionnaire JHPIEGO. Translated into Amharic Multiple regression with poor or good participation and other factors. Frequencies of husbands who participated in various BPCR activities.	See Table 3	1. †
Ghana						
Atuahene, Arde-Acquah, Atuahene, Adjuik&Ganle (2017) [32]	To describe the level of male involvement in inner city safe motherhood projects	Married men aged 18+ whose wife/partner was pregnant and in 3rd trimester or had children 5 or younger Average age 37 (256)	Multistage sampling procedure to select: houses, households then respondents. Simple random sampling.	- Cross-sectional observational study -Interviewer administered study-designed questionnaire: socio demographic variables, ANC attendance, birth processes. Piloted in similar region. -Descriptive statistics	See Table 3	.68†
Kenya						
Dunn, Haque&Innes (2011) [33]	To assess men’s awareness of danger signs of obstetric complications	Men with wife or partner who had undergone childbirth in preceding 36 months Average (SD) age 35 (8), 41% 0–2 children, 98% Christian (167)	Purposively sampled for education diversity	- Cross-sectional observational study -Study specific questionnaire identifying dangers signs as true or false - Descriptive statistics	See Table 3	.45†
Nigeria						
Oguntunde et al. (2019) [34]	To assess the determinants of MP knowledge of danger signs in pregnancy	Married men with at least one wife younger than 25 years (1627)	Multistage random selection	- Cross-sectional observational study -Interviewer administered standard JHPIEGO questionnaire -Multivariate analysis	See Table 3	.90†
Sekoni (2014) [35]	To assess MP knowledge of obstetric danger signs	Men aged 15–65 with at least one child < 3 years (259)	Multistage random selection	- Cross-sectional observational study -Interviewer administrated structured questionnaire -Descriptive statistics	See Table 3	.63†
Rwanda						
Kalisa&Malande (2016) [36]	To assess level of male partner involvement in birth plan, attitude of women towards BPCR	Pregnant women and MP presenting as referrals to health service 59% completed primary education, 94% married, average age 27 (women) and 31 (MP) (193 women + 203 MP)	Purposive sampling for referrals to health service. Healthcare workers recruited participants	- Cross-sectional observational study -Pre-tested structured interview questionnaire based on ‘Monitoring BPCR WHO’. Adapted for local conditions. Socio demographic characteristics, medical history, reason for referral, level of male partner’s involvement, women’s attitudes towards male involvement in BPCR, BPCR. - Frequencies, chi-square test. Bivariate logistic regression. Multivariable logistic regression.	See Table 3	.82†
Tanzania						
August, Pembe, Mpembeni, Axemo & Darj (2015) [37]	To assess men’s knowledge of danger signs and BPCR	Men with partners who gave birth within past 2 years (756)	Two stage sampling procedure. All health facilities listed, then ballot to identify 14. Two villages within the catchment population for the facilities randomly selected	- Cross-sectional observational study -Standardised structured questionnaire by JHPIEGO adopted for Tanzania context. Socio demographic, attended ANC, experienced complications, knowledge of danger signs -Descriptive statistics and logistic regression	See Table 3	1. †
Shimpuku, Madeni, Horiuchi&Leshabari (2017) [38]	To assess predicted birthplace intentions	Pregnant women ≥16, (no psychological or physical illness), husbands and family members ≥16living with women (121: 42 pregnant women, 35 husbands and 44 family members)	Non-probability sampling and purposive sampling via village leaders for pregnant women	- Cross-sectional observational study −38 item study-specific birth intention questionnaire -Chi-square test, ANOVA, multiple regression, correlation	See Table 3	.59†
Uganda						
Kakaire, Kaye & Osinde (2011) [39]	To assess factors associated with BPCR & level of male participation in the birth plan among emergency obstetric referrals	Pregnant women admitted as emergency obstetric referrals Average for women and men: age 26, 32, 73 and 55% had no or primary education, 81% married (140)	Purposive sampling for referrals Healthcare workers recruited participants	**-** Cross-sectional observational study **-**Questionnaire: Socio-demographic, medical history, Birth preparedness, roles of spouses in birth plan -Medical records: obstetric complications, reasons for referral, obstetric care obtained at the referral and referring sites and availability of a birth plan - Frequencies, Chi-square test. Bivariate logistic regression. Multivariable logistic regression.	-Men’s responsibility to save money for birth and organise transport. However results showed 44% of women used their own money for birth	.77†
**Mixed methods research** ***n*** **= 5603**						
Ethiopia						
Andarge et al. (2017) [40].	To assess the factors influencing BPCR among pregnant women in Ethiopia	Pregnant women and their partners (707) women and 6 FGD with male partners	Multistage sampling	- Cross-sectional observational study, FGD -Interviewer administered standardised JHPEIGO questionnaire	Qualitative findings: Male FGD participants agreed that main BPCR practice was saving money and preparing special birth porridge Quantitative data NR for men	.85*
Malawi						
Aarnio, Chipeta&Kulmala (2013) [41]	To explore husbands’ perception of birth care	Ever married men whose wives had been pregnant in the last 5 years - Median age 33, majority Islamic, 67% literate, 99.5% married, 10% polygamous, majority 1–3 children, 98% male breadwinner, majority access to health facility by walking, bicycle, or public transport. (389)	Systematic random sampling, first eligible person in household interviewed	- Cross-sectional observational studyincluding some open-ended questions -Study-specific questionnaire. Closed- and open-ended questions about men’s perceptions of and involvement in antenatal care, birth preparedness, choice of birth place, obstetric complications, birth care and postpartum care. Picture cards of 5 danger signs; asked if they would go to hospital with these issues. -Descriptive statistics - Open-ended questions with narrative with content analysis.	MP make decisions about MCH decisions and BPCR MP often seek help at a facility for danger signs (except convulsions)	.73*
Nigeria						
Iliyasu, Abubakar, Galadanci&Aliyu (2010) [42]	To assess BPCR and male involvement	Ever married men whose wives had ever been pregnant, and their wives and community leaders - Majority Muslim, aged 20–39, employed including government, farmers and private employees; 70% had some education. (389)	Multistage systematic sampling of households	- Cross-sectional observational study, IDI -Standardised structured questionnaire by JHPIEGO: Demographic, perception of high risk pregnancy and danger signs during pregnancy, birth preparedness and complication readiness,) participation of men and spousal attitudes towards these issues. IDI guide for community leaders: reasons for low participation of men in maternity care Descriptive statistics. Chi-square test. Multivariate logistic regression Thematic analysis, illustrative quotes	See Table 3	.95†
Nwakwuo&Oshonwoh (2013) [43]	To assess MP level of involvement in perinatal health events	Men whose spouses had children or had maternal event last 1 year and local resident - Average age 38, majority married, educated to secondary, Christians, public servants (386, 20 IDI)	Multistage sampling technique for survey. Houses numbered then systematically selected. Ballot used to identify household if more than one in dwelling. All eligible men in household approached to be involved Purposive sampling for antenatal woman or postnatal woman.	- Cross-sectional observational study, IDI -Study-specific questionnaire including open and closed questions Interview guide on topic of knowledge and attitudes of wives to husband involvement. Descriptive statistics. Chi-square test and Fisher’s exact test. No further details given.	See Table 3	.68†
Odimegwu, Adewuyi, Odebiyi, et al. (2005) [44]	To examine the role of men in emergency obstetric care	(1957 women and 1720 MP)	Random selection from study drafted household list	- Cross-sectional observational study, FGD, -Topic guide on pregnancy complications and role of MP -Thematic analysis -Multivariate	-Men aware obstetric conditions particularly in relation to pregnancy signs and labour pains (53.2%). -Men perform important tasks during obstetric conditions (89.2%).	.54†
Uganda						
Singh, Lample& Earnest (2014) [45]	To understand men’s participation in MCH, and’ men’s and women’s views on increased male partners’ involvement	Women who were pregnant or gave birth in last 1–3 years and MP, and key informants Religion Christian or Muslim depending on village, majority married, majority 18–28 years old, majority 1–2 or 3–7 children. (35): 23 women and 12 men	Purposive and opportunistic sampling through key informants	- Cross-sectional observational study, FGD -Study specific self-report questionnaire for men and women Topic guide for FGD: birth preparations, ANC, health services, involvement of men and factors impacting pregnancy and labour Thematic analysis with triangulation between FGD and questionnaire data	MP involved in pregnancy decisions Women said money was a challenge in birth preparation Money for birth is responsibility of MP Men and women agreed need to improve MP involvement	.60*

*MP* Male partner, *FGD* Focus group discussions, *IDI* In-depth interview, *SSI* Semi-structured interview, *SBA* Skilled birth attendant, *HW* Health worker, *TBA* Traditional birth attendant, *ANC* Antenatal care, *MCH* Maternal child health, *QA* Quality assessment max score 1, *JHPIEGO* John Hopkins Program for International Education in Gynaecology and Obstetrics, *MP* Male partner, * Kmet qualitative checklist, † Kmet quantitative checklist

**Table 2:** Intervention studies included

Interventional studies ***n*** = 1983 and 12 FGD								
Authors (date)	Country & Study design	Aim	Inclusion criteria and sample (n)	Intervention details & duration	Sampling and recruitment strategy	Data source & analysis	Findings	QA
Nigeria								
Ibrahim et al. (2014) [46]	Nigeria: Quasi-experimental study	To assess the effect of a health promotion intervention on MP involvement in BPCR	Married men whose wives had been pregnant in preceding 3 years (205 pre and 206 post)	*Behavioural intervention* Five interactive workshops. A film shown and discussion. Almanacs with messages of MP involvement and reproductive health	Multistage random sampling of intervention and control group	Standardised survey and qualitative interviews with all participants. -Pre-post surveys analysed. Qualitative data thematically analysed.	No increase or change in BPCR following intervention. Qualitative analysis revealed religious beliefs prohibited BPCR	.45†
Tanzania								
Mushi, Mpembeni & Jahn (2010) [47]	Tanzania: Quasi experimental	To develop, test and assess safe motherhood intervention	Pregnant women and their partners Age 19–53, median 29. 62% married, most married by 18 years old, 41% never been to school. 94% Muslim (242:153 women, 69 partners	*Safe Motherhood Intervention* Home visits with pregnant women and husband and key community members about danger signs, complications, BPCR, ANC, and birth with a skilled attendant	Random sample of residents in four villages pre and post intervention	Questionnaire: demographic, attendance ANC, risk factors, referral status, place of birth. Qualitative: iSSI with closed and open-ended questions. Referral information. -Outcomes compared pre and post.	-No significant differences in MP knowledge of danger signs between pre and post intervention -MP awareness of: 3 risk practices during pregnancy pre 58 (58%) vs post 39 (55%); 3 danger signs during pregnancy pre 54 (54%) vs 42 (60.9%); 3 complications during delivery pre 41 (41%) vs post 36 (52.2%); 3 practices that contribute to delay in seeking care pre 52 (52%) vs post 40 (58%); MP who did not believe pregnancy complications are due to non-observance of tradition pre 36 (36%) vs 40 (58%).	.67†
August, Pembe, Mpembeni, Axemo & Darj (2016) [48]	Tanzania: Quantitative pre/post quasi experimental	To evaluate the Home Based Life Saving Skills in terms of male knowledge of danger signs, joint decision making, birth preparedness and attending ANC	Men with partners who gave birth in last 2 years (1426)	*Home Based Life Saving Skills* -Joint training of pregnant women and family Aim to educate about BPCR, danger signs, promote health seeking behaviour and provide skills to handle emergencies Teaching through checklists, skill acquisition and Take Action cards Four home visits	Two-stage cluster sampling, random sampling of villages with all eligible men approached. -intervention and comparison group	Standardised JHPIEGO questionnaire Descriptive statistics, net intervention effect difference between baseline and endline in intervention minus effect in comparison group	Outcomes reported as Net Intervention Effect (NIE): Effect of the intervention on male involvement: −3+ danger signs during pregnancy, 3+ during childbirth, and 3+ during postpartum NIE = 27% (CI: 15.3–38.5; *p* < 0.001). -MP who made three or more BP/CR actions increased significantly, NIE = 26.8%, CI: 15.3–38.2; *p* < 0.001).	1. †
Uganda								
Ekirapa-Kiracho et al. (2016) [49]	Uganda: Evaluation study FGD, IDI	To reflect on gains, challenges and lessons learnt from working with communities to improve maternal and newborn health in rural Uganda	Women who recently gave birth and their partners Community stakeholders (20 IDIs & 12 FGDs)	*Participatory Action Research* MANIFEST (maternal and neonatal implementation for equitable systems) Aim to increase maternal and newborn health through community awareness Intervention: diagnose problem, plan action, take action, learn from action	Not described	Topic guide not described Thematic analysis	-No quantitative analysis of MP BPCR changes -Men and women anecdotally reported increased awareness about BPCR -Alternative communication strategies are needed to reach men outside the minority who were involved in home visits and community meetings -Some changes were observed among men following intervention, e.g. increased support via nutritious diets, purchasing birth items, and saving for childbirth said women during FGDs.	.60*

*MP* Male partner, *FGD* Focus group discussions, *IDI* In-depth interview, *SSI* Semi-structured interview, *SBA* Skilled birth attendant, *HW* Health worker, *TBA* Traditional birth attendant, *ANC* Antenatal care, *MCH* Maternal child health, *QA* Quality assessment max score 1, *JHPIEGO* John Hopkins Program for International Education in Gynecology and Obstetrics, *MP* Male partner, * Kmet qualitative checklist, † Kmet quantitative checklist, *NIE* Net Intervention Effect (difference between baseline and endline in intervention minus comparison group)

### Extent and quality of research

The identification of papers is illustrated in Fig. 1. The extent and quality of research has been summarised below using the sub-headings: Study designs, populations and geographic locations and quality of research.

### Study designs of included research

There were 35 studies included, comprising: 13 qualitative, 13 quantitative (cross-sectional), 5 mixed methods, and 4 intervention studies. Research methods included: focus group discussions (total participants approximately *n* = 602); in-depth interviews (*n* = 393); cross-sectional surveys (*n* = 5942); mixed methods (*n* = 5603) and intervention studies (*n* = 1983).

### Populations and geographic locations of included research

Overall data were reported from approximately 14,550 participants including pregnant women or those who had experienced pregnancy or childbirth within the previous 3 years, their male partners and key informants such as health workers and community leaders.

Studies took place in: Burkina Faso (1); Ethiopia (5); Ghana (4); Kenya (2); Malawi (3); Nigeria (6); Tanzania (6); Uganda (6), Zambia (1) and Rwanda (1). The majority of studies reported exclusively on research in rural areas, with the remaining studies reporting on research in either urban areas or a mixture of urban and rural settings.

### Quality of research overall

The study designs included in the review reflect an emerging field of research. The majority of study designs were either qualitative or cross-sectional observation surveys (many purely descriptive). There were no randomised controlled trials, but there were three quasi-experimental intervention studies [46–48] and one evaluation study [49]. These study designs limit the level of evidence available on the topic.

Sixteen primarily qualitative research studies were assessed using the Kmet qualitative checklist (see column QA in Tables 1 and 2). The quality of the studies was reasonable with a median score of .75 (range of .60 to .85). The criteria most commonly not met were the use of verification procedures to establish credibility and reflexivity of account (no study adequately documented the latter).

Fifteen primarily cross-sectional studies were assessed using the Kmet quantitative checklist (see column QA in Tables 1 and 2). The quality for the sources of evidence was reasonable with 3 studies receiving full scores [1] and only four dropping below .6. They produced a median score of .76 (range of .45 to 1). The criteria most commonly not met included: not controlling for confounding variables, not providing a measure of variance and flaws relating to the outcome measure.

Of three intervention studies included in this review, the median score for the Kmet quantitative checklist, was .45 (range: .67–1.0). Only one completed appropriately complex analysis of the data. A fourth evaluation study was assessed using the qualitative checklist to fit the reported data; this received a score of .60.

### Conceptualisation of male partner involvement in BPCR

Nine studies discussed the role or conceptualisation of male partner involvement in BPCR using qualitative research methods [9, 19–21, 27, 36, 39, 41, 45]. Qualitative studies did not report a structured definition of BPCR; instead they relied on participants’ accounts of preparing for birth.

Male partners were described as playing an important role in pregnancy and birth decisions including preparing for birth and potential complications [27, 45]. These processes were complex, involved many people and varied between communities.

It was reported that although women have a central position in pregnancy and birth, they frequently lack decision-making power and resources [45]. Several studies described scenarios where pregnant women lacked agency and were not participants in decision-making processes around their health and body [16, 19, 22, 23]. This pertained in particular to decision about when to attend a health facility for a normal birth or to seek help in the case of obstetric complications.

Specific BPCR responsibilities reported for male partners were often related to material support [16, 20, 22, 40, 41, 45]. The most common role for men was to provide financial support for buying birth items (for example, a birth kit) or providing nutritious food [40]. Another common role described was to identify and organise transport to a facility [18, 19, 21].

A common conceptualisation was that male partners viewed pregnancy and childbirth as a “natural” process and this then influenced their ideas of how to prepare for birth [22]. This was explained through the use of finances for birth, which would not be used for birth in a facility unless complications occurred, and would instead be used for clothes and food. In general male partners strived to do their best [26], for example providing adequate care to one’s wife was considered a symbol of social status in Malawi [41]. However, men’s involvement was often hampered by barriers. Lack of awareness and poverty were common challenges experienced by male partners in fulfilling their perceived responsibilities [16, 40].

### Measurement of BPCR in male partners

Fourteen studies reported the level of BPCR or recognition of obstetric danger signs among male partners [30–32, 36–38, 41–43, 49]. All except one study [49] used quantitative methods. Nine studies employed a standardised tool developed by John Hopkins Program for International Education in Gynecology and Obstetrics (JHPIEGO) to measure BPCR (this consisted of using the BPCR tool to identify if male partners had performed the following items: saving money for birth; identifying transport; identifying the birth location; knowing the signs of pregnancy complications; identifying a skilled birth attendant, identifying someone to donate blood) or measured the recognition of pregnancy danger signs (please see Table 4 for a full list) [28–31, 34, 35, 37, 42, 43]. Each study adapted the questionnaire to local conditions. Three studies used a study specific questionnaire to measure BPCR [38, 41, 43]. Two studies reported aspects of BPCR through a questionnaire designed for other purposes [32, 36].

As displayed in Table 3, proportions of male partners who had completed each BPCR indicator varied between studies. Commonly performed actions included: saving money, 20–99%; purchasing a birth kit, 38–54%; organising transport, 10–69%; and identifying where to go in an emergency, 2–78%. The least commonly performed actions included: identifying a skilled birth attendant, 1–41% and identifying a blood donor, 0–18%.

**Table 3:** Rate of BPCR among male partners

Standardised measurement of BPCR n (%)							Study specific measurement of BPCR n (%)			
Birth preparedness and complication readiness	Tanzania (*n* = 756) [37]	Ethiopia (*n* = 824) [30]	Ethiopia (*n* = 592) [31]	Nigeria (*n* = 382) [42]	Ethiopia (*n* = 406) [28]	Ethiopia (*n* = 398) [29]	Ghana (*n* = 256) [32]	Rwanda (*n* = 396) [36]	Tanzania (*n* = 121) [38]	Malawi (*n* = 389) [41]
Identified birth kit	394 (54.3%)	309 (37.5%)	NR	NR	354 (86.5%)	301 (80%)	NR	NR	NR	NR
Saved money for healthcare/ emergency	342 (47.2%)	218 (26.5%)	446 (75.3%)	76 (19.5%)	158 (39.6%)	287 (76.3%)/ 215 (57%)	254 (99%)	NR	62.9%	NR
Identified transport	74 (10.2%)	91 (11%)	357 (60.3%)	94 (24.2%)	178 (44.6%)	246 (65.4%)	NR	242 (69%)	NR	99%
Identified birthplace/ where to go in emergency	13 (1.8%)	25 (3%)	437 (73.8%)	NR	218 (54.6%)	234 (62.2%)	NR	NR	NR	82.9%
Identified skilled birth attendant	6 (0.8%)	67 (8.1%)	242 (40.9%)	24 (6.2%)	117 (29.3%)	123 (32.7%)	NR	NR	NR	NR
Identified blood donor	1 (0.1%)	3 (.4%)	108 (18.2%)	3 (.8%)	190 (47.6%)	65 (17.3%)	NR	NR	NR	NR
Made no preparations	205 (28.3%)	256 (31.1%)	NR	NR	NR	NR	NR	NR	NR	NR
Made one preparation	(43.7%)	363 (44.1%)	NR	NR	NR	NR	NR	NR	NR	NR
Made at least three preparations	87 (11.2%)	82 (9.9%)	NR	NR	NR	NR	NR	NR	NR	NR
Made five or more preparations	NR	NR	324 (54.7%)	NR	187 (46.9%)	227 (60.4%)	NR	NR	NR	NR

*NR* Not reported

As displayed in Table 4, there was variability in the way studies reported men’s recognition of various pregnancy, childbirth and postpartum danger signs. Pregnancy danger signs were more commonly reported indicators (compared to childbirth or postpartum). Several studies reported that very small proportions of male partners indicated recognition of danger signs (< 15% recognition for most indicators) [30, 35, 37, 42]. The remaining studies reported reasonably low rates (< 60% for most indicators) [28, 31, 34, 43].

**Table 4:** Male partners’ knowledge of danger signs

MP knowledge of Danger signs n (%)								
Obstetric danger signs	Tanzania (*n* = 756) [37]	Ethiopia (*n* = 824) [30]	Ethiopia (*n* = 592) [31]	Nigeria (*n* = 389) [42]	Ethiopia (*n* = 406) [28]	Nigeria (*n* = 386) [43]	Nigeria (*n* = 1627) [34]	Nigeria (*n* = 259) [35]
**During pregnancy**								
High fever	157 (21.6%)	105 (12.7%)	NR	16 (4.1%)	NR	233 (60.4%)	NR	NR
Severe abdominal pain	107 (14.7%)	97 (11.8%)	NR	NR	NR	NR	NR	NR
Excessive vaginal bleeding	79 (10.1%)	94 (11.4%)	NR	202 (51.9%)	213 (53.4%)	226 (58.9%)	NR	30 (11.6%)
Abnormal body movements	70 (9.6%)	61 (7.4%)	NR	60 (15.4%)	NR	199 (51.6%)	NR	NR
Severe headache	61 (8.4%)	70 (8.5%)	NR	35 (9.0%)	NR	230 (59.6%)	NR	NR
Swollen hands face	22 (3%)	59 (7.2%)	NR	65 (16.7%)	165 (41.4%)	219 (56.7%)	NR	NR
Loss of consciousness	15 (2.1%)	33 (4%)	NR	129 (33.2%)	NR	NR	NR	NR
Blurred vision	6 (.8%)	13 (1.6%)	NR	36 (9.3%)	117 (29.3%)	NR	NR	NR
Paleness	NR	NR	NR	NR	NR	206 (53.4%)	NR	NR
**During childbirth**								
Excessive vaginal bleeding	165 (22.7%)	102 (12.4%)	NR	NR	264 (66.2%)	150 (38.9%)	NR	NR
Convulsions	101 (13.9%)	54 (6.5%)	NR	NR	163 (40.9%)	22 (5.7%)	NR	NR
Retained placenta	65 (9%)	37 (4.5%)	NR	NR	120 (30.1%)	214 (55.4%)	NR	26 (10.0%)
High fever	42 (5.8%)	98 (11.9%)	NR	NR	NR	NR	NR	NR
Prolonged labour	35 (4.8%)	85 (10.3%)	NR	NR	169 (42.4%)	NR	NR	NR
Severe headache	29 (4%)	64 (7.8%)	NR	NR	NR	NR	NR	NR
Loss of consciousness	13 (1.8%)	29 (3.5%)	NR	NR	NR	NR	NR	NR
**During postpartum**						NR		
Excessive vaginal bleeding	129 (17.8%)	105 (12.7%)	NR	NR	137 (34.3%)	NR	NR	NR
High fever	52 (7.2%)	99 (12%)	NR	NR	84 (21.1%)	NR	NR	NR
Abnormal body movements	37 (5.1%)	56 (6.8%)	NR	NR	NR	NR	NR	NR
Loss of consciousness	10 (1.4%)	31 (3.8%)	NR	NR	NR	NR	NR	NR
Foul smelling discharge	8 (1.1%)	74 (8.9%)	NR	NR	64 (16%)	NR	NR	33 (12.7%)
Severe headache	6 (.8%)	78 (9.5%)	NR	NR	NR	NR	NR	NR
**Summary**	NR	NR	NR	NR	NR	NR	NR	NR
One sign during pregnancy	389 (53.7%)	407 (49.4%)	250 (42.2%)	NR	NR	NR	NR	NR
Two signs during pregnancy	NR	NR	NR	NR	NR	NR	(507) 63.7%/ (352) 42.4%	NR
One sign during childbirth	318 (43.9%)	271 (32.9%)	NR	NR	NR	NR		NR
Two signs during childbirth	NR	NR	NR	NR	NR	NR	(570) 71.7%/ (516) 62.1%	NR
One sign postpartum period	251 (34.6%)	213 (25.8%)	NR	NR	NR	NR		NR
Two signs postpartum period	NR	NR	NR	NR	NR	NR	(429)53.9%/ (290) 34.9%	NR

### Conceptualisation of male partner involvement in responding to obstetric complications

Eleven studies reported information about male partners role in responding to complications [16–20, 22–26, 41]. All, but one study, were qualitative [41].

In all descriptions of families responding to complications, male partners were central to the decision making process [16–20, 22–26, 41]. Two studies reported that male partners were involved in maternal healthcare only when pregnancy complications occurred [16, 17]. Frequently the decisions male partners made in relation to preparing for birth were not unilateral, but involved consultation with women, other family members such as male partners’ mothers or traditional birth attendants [16, 19, 27, 41].

The conceptualisation of complications in childbirth by study participants was a mixture of medical and spiritual conditions. Responses were guided by these ideas, some conditions requiring medical treatment and others needing guidance from a traditional birth attendant (TBA). For example in one study male partners would seek advice from a spiritual healer instead of a health facility if their partner experienced convulsions while pregnant [22].

In general, male partners were found to be involved throughout the process of responding to complications. In the healthcare setting there were reports that male partners were not always welcomed. Some men described being excluded by health professionals or not having a clear role [18, 24, 26]. In the home setting partners were responsible for sourcing and administering several types of medications [25]. Emotionally male partners were impacted by the experience of pregnancy complications with experiences characterised by intense fear, worry and loss [24].

It was noted that although male partners often played a key role in decisions around maternal health, they lacked knowledge on maternal and child health [20, 22, 40]. Both women and men reported a desire for men to be more knowledgeable about BPCR [22].

### Interventions to improve BPCR or knowledge of danger signs among male partners

The findings from four trials of interventions to improve levels of male partner knowledge of BPCR or awareness of danger signs are summarised in Table 2 Evaluation methods were mixed in the complexity of analyses and type of data collected - which prohibits reliable comparison. The most methodologically robust evaluation did report a significant improvement in male partners’ awareness of danger signs [48]; the remaining three did not report any improvement.

## Discussion

To our knowledge, this is the first scoping review conducted of sub-Saharan studies on the involvement of male partners in BPCR and response to obstetric emergencies. The synthesis of the literature on this topic has implications for research, policy and practice across the region.

### Extent and quality of research into male partner involvement in BPCR

The diversity of study designs, aims and source countries in this body of literature reflects an emerging stage of research; as a result, the review yielded strong evidence in some areas and gaps in others. The evidence from quantitative studies may be regarded as limited because most of the study designs sit low on the hierarchy of evidence [50] and the samples/studies do not represent all of the Sub-Saharan Africa. The studies are limited to certain regions within the countries studied and lack population level results. There is a distinct lack of higher-level research, for example robust research trialling interventions to increase male involvement in BPCR. Although the average quality of included studies was reasonable, it is not possible to generalise based on the large proportion of qualitative research.

However, for research at an emergent stage, the evidence did contain some strengths. One of the strengths is the inclusion of several studies using qualitative methods by local researchers (often in local dialects). This suggests there has been an effort to conceptualise male partner involvement in BPCR in a way that is culturally sensitive and does not impose an inappropriate framework. The development of a standardised tool to measure BPCR [6] and the use of this tool by the majority of quantitative research projects supports inter-study comparisons, and its adaptation to local contexts ensures culturally appropriateness. The quality assessment revealed most studies were performed with sound methodology and collected data from over 13,000 people. The findings yielded relatively consistent results, despite diverse methods and contexts.

### Conceptualisation of male involvement in BPCR and responding to obstetric emergencies

Defining the role of or conceptualising male partner’s involvement in preparing for birth and responding to complications was achieved using qualitative research methods. This is appropriate given the lack of adequate evidence in this area. However qualitative research often has small, selective samples and cannot be used to generalise to the broader populations. Moreover, the geographic coverage of the research was inconsistent across Sub-Sahara Africa, leaving many countries and cultural groups unrepresented and some countries with multiple studies. Consequently, the concepts and roles of male partners’ involvement in BPCR can only be used as a guide to inform future research and cannot be considered definitive.

The conceptualisation of male partner involvement yielded key themes across studies. In many contexts male partners had some role in preparing for birth and consistently across studies they were instrumental in responding to obstetric complications. They were directly involved through their position in the chain of decisions and indirectly involved through providing financial and logistical support. The understanding of their role is significant to the development of interventions to support BPCR in families and communities. Knowledge of the male partner’s role should be further developed with more widespread research, to inform interventions to increase BPCR in families.

Male partners were interested in taking appropriate action [26]. However, their involvement did not always align smoothly with other systems and processes. Participants reported difficulties in dealing with the healthcare system, with some male partners reporting being excluded from healthcare decisions [39]. On the other hand, healthcare staff described delays in providing care because male partners were not available to give permission. Poverty was also described as a common barrier to male partners fulfilling what they saw as their responsibilities [45].

### Measurement of male partner’s involvement in BPCR

Measurement of male partner’s level of BPCR had some degree of consistency because many studies used the tool developed by JHPIEGO [6]. However, there were still discrepancies between studies in scoring or summarising the results from the tool (e.g. defining good/poor BPCR). Although there has been an effort to validate and adapt the standardised tool to different cultural contexts, it is not clear if it has been adapted for use with male partners.

Studies that did not use the tool developed by JHPIEGO contained diverse definitions of BPCR, making it hard to compare. The studies using research specific tools to measure involvement also tended to report higher levels of involvement.

There was little consistency in summarising recognition of danger signs: most studies had unique indicators, which prevented a comparison between studies. This variance was greater between studies not using a standardised measure. For example it was reported that 58% of participants could correctly identify more than *nine* danger signs when presented and asked if they were ‘true’ or ‘false’ [33] with a study specific questionnaire. Researchers using a standardised measure asked men to spontaneously mention danger signs and found that 42% (37), 49% (30) and 53% (31) of men could *not* identify more than *one*.

The literature overall presented a general theme that although male partners frequently make decisions about maternal healthcare, in general, they lack knowledge regarding maternal health concerns. This was described in the findings from both qualitative and quantitative papers included in this scoping review. Male partners’ recognition of pregnancy and birth danger signs was poor across all studies and their level of BPCR was generally low. Saving money and purchasing birth items were the most commonly performed actions, and some BPCR actions were almost completely neglected. For instance, very few men identified a potential blood donor or a skilled birth attendant. This increases the risk of adverse pregnancy and birth outcomes. These results suggest that male partner’s preparation for birth and complications can often be improved, and this has the potential to improve outcomes for women and children.

### Interventional studies

Studies of male involvement interventions were the least common type of research with large variation in the quality of methods. Only one interventional study was considered robust and received the maximum QA score of 1 [48]. *Home Based Life Saving Skills* provided joint training of pregnant women and family with the aim to educate about BPCR, danger signs, promote health seeking behaviour and provide skills to handle emergencies. This study reported significant improvement in male partners’ recognition of pregnancy danger signs, suggesting that properly designed interventions may be useful in improving male partner knowledge.

### Implications for future research

The gaps revealed in this scoping review provide future research opportunities. In many sub-Saharan African countries, there is inadequate evidence on the role of male partners in preparing for birth and responding to pregnancy complications. From the 46 countries listed as Sub-Sahara Africa by the United Nations, only 10 were identified as having any evidence regarding male involvement in BPCR. Future research could focus on reporting levels of BPCR among male partners in Sub-Saharan countries with long delays in receiving care not represented in this review.

From the countries already represented (for example Ethiopia), future research could focus on examining associations between male partner’s level of BPCR and maternal health outcomes. It is assumed because of male partners’ role in decision making that increased knowledge of danger signs and BPCR will translate into improved maternal health outcomes [4], but this hypothesis remains to be tested in different contexts.

Research should also focus on evaluating interventions to improve male partner level of BPCR and knowledge of obstetric complications. This is extremely limited at present and it is not clear what interventions are useful in improving male partner BPCR and knowledge of danger signs.

### Implications for policy, programs and practice

The findings suggest that in specific countries in sub-Saharan Africa, male partners are involved in BPCR and responding to pregnancy complications, yet their level of preparation and knowledge of pregnancy complications is poor. Policies, programs and practice could focus on improving male partners’ level of knowledge about complications and the importance of preparing for birth. As with all interventions encouraging male involvement in maternal health, this would need to be performed in a way that did not compromise women’s autonomy or safety and may involve additional training for healthcare workers.

## Conclusions

In conclusion, the diversity of study designs, aims and source countries in this body of literature reflects an emerging stage of research; as a result, the review yielded strong evidence in some areas and gaps in others. Male partners’ involvement in BPCR and responding to obstetric emergencies can be conceptualised as being centrally involved in responding to complications and having some role in preparing for birth through their position in the chain of decisions and provision of logistic support. However, their knowledge of pregnancy complications and level of preparation for birth is low, suggesting they are making decisions without being fully informed. There is limited evidence on interventions to improve men’s knowledge on BPCR and signs of complications, however improvements were recorded following an intervention in Tanzania [48]. Future research efforts should be focused on producing standardised, culturally appropriate, higher level evidence and randomised controlled trials of interventions. As pregnancy complications are a leading cause of maternal mortality in Sub-Saharan Africa, appropriate preparation for birth and complications by women, male partners, families and the community have the potential to lower these risks.

## Supplementary Information

**Additional file 1.** Contains a supplementary table documenting the search strategy.

## Data Availability

All publications referred to in this review are publicly available.

## Abbreviations

BPCR: Birth preparedness and complication readiness
WHO: World health organisation
DHS: Demographic and health survey
MP: Male partner
FGD: Focus group discussions
IDI: In-depth interview
SSI: Semi-structured interview
SBA: Skilled birth attendant
HW: Health worker
TBA: Traditional birth attendant
ANC: Antenatal care
MCH: Maternal child health
QA: Quality assessment
JHPIEGO: John Hopkins Program for International Education in Gynaecology and Obstetrics
*: Kmet qualitative checklist
†: Kmet quantitative checklist
QA: Quality assessment max score 1
