# Supplementary material for: Male partner involvement in birth preparedness, complication readiness and obstetric emergencies in Sub-Saharan Africa: a scoping review
Source: BMC Pregnancy Childbirth. 2021 Feb 12;21:128. doi: 10.1186/s12884-021-03606-x (PMC7881528; doi:10.1186/s12884-021-03606-x)
Supplement: Supplementary file 1 — Additional file 1. Contains a supplementary table documenting the search strategy. [file 12884_2021_3606_MOESM1_ESM.pdf]

Supplementary Table Search strategy

Concept	Database	Mesh (different each database)	Keywords (same each database)
Male involvement	Embase	spouse/ or domestic partner/ or husband/ or wife/	Male* involve* or male* participat* or male attend* or male* engag* or husband* involve* or husband* participat* or husband* attend* or husband* engag* or spouse* involve* or spouse* participat* or spouse* attend* or spouse* engag* or father* involve* or father* participat* or father* attend* or father* engag* or paternal involve* or paternal participat* or paternal attend* or paternal engag*
	MEDLINE	paternal behavior/ or spouses/ men/ or sexual partners/ or spouses/	
	Maternity and infant care	Male or Partners or Fathers or Participation	
Birth preparedness and complication readiness	Embase	childbirth education/ or prenatal care/	Birth preparedness or complication readiness
	MEDLINE	Prenatal Care/	
	Maternity and infant care	Pregnancy or Birth	
Obstetric emergencies	Embase	obstetric delivery/ or obstetric emergency/ or obstetric hemorrhage/ pregnancy complication/ pregnancy/ or reproduction/ or adolescent pregnancy/ or first trimester pregnancy/ or/ mother fetus relationship/ or multiple pregnancy/ or second trimester pregnancy/ or third trimester pregnancy/ or unplanned pregnancy/ or unwanted pregnancy/ or childbirth/	emergenc* or complication* or obstetric or maternal near miss or maternal mortality or obstetric morbidity or delivery or safe motherhood or haemorrhage
	MEDLINE	Pregnancy Complications/ Pregnancy/ or Delivery, Obstetric/ or Obstetric Labor Complications/	
	Maternity and infant care	Complications	
Sub-Saharan Africa	Embase	africa south of the sahara"/ or africa/ or angola/ or benin/ or botswana/ or burkina faso/ or burundi/ or cameroon/ or cape verde/ or central africa/ or central african republic/ or chad/ or comoros/ or congo/ or cote d'ivoire/ or democratic republic congo/ or djibouti/ or equatorial guinea/ or eritrea/ or ethiopia/ or gabon/ or gambia/ or ghana/ or guinea/ or guinea-bissau/ or kenya/ or lesotho/ or liberia/ or madagascar/ or malawi/ or mali/ or mayotte/ or mozambique/ or namibia/ or niger/ or nigeria/ or rwanda/ or sahel/ or senegal/ or sierra leone/ or somalia/ or south africa/ or south sudan/ or sudan/ or swaziland/ or tanzania/ or togo/ or uganda/ or zambia/	

		or zimbabwe/	
	MEDLINE	africa south of the sahara"/ or africa, central/ or africa, eastern/ or africa, southern/ or africa, western/ africa/ or cameroon/ or central african republic/ or chad/ or congo/ or "democratic republic of the congo"/ or equatorial guinea/ or gabon/ or "sao tome and principe"/ or burundi/ or djibouti/ or eritrea/ or ethiopia/ or kenya/ or rwanda/ or somalia/ or south sudan/ or sudan/ or tanzania/ or uganda/ or angola/ or botswana/ or lesotho/ or malawi/ or mozambique/ or namibia/ or south africa/ or swaziland/ or zambia/ or zimbabwe/ or benin/ or burkina faso/ or cabo verde/ or cote d'ivoire/ or gambia/ or ghana/ or guinea/ or guinea-bissau/ or liberia/ or mali/ or mauritania/ or niger/ or nigeria/ or senegal/ or sierra leone/ or togo	
	Maternity and infant care	Developing countries or Africa	

.
